# SARS-CoV-2 Seroprevalence Studies in Pets, Spain

**DOI:** 10.3201/eid2906.221737

**Published:** 2023-06

**Authors:** Sandra Barroso-Arévalo, Lidia Sánchez-Morales, Jose A. Barasona, Lucas Domínguez, José M. Sánchez-Vizcaíno

**Affiliations:** VISAVET Health Surveillance Center, Madrid, Spain (S. Barroso-Arévalo, L. Sánchez-Morales, J.A. Barasona, L. Domínguez, J.M. Sánchez-Vizcaíno);; Complutense University of Madrid, Madrid (S. Barroso-Arévalo, J.A. Barasona, L. Domínguez, J.M. Sánchez-Vizcaíno)

**Keywords:** COVID-19, SARS-CoV-2, severe acute respiratory syndrome coronavirus 2, viruses, respiratory infections, seroprevalence, Spain, pets, zoonoses, epidemiology

## Abstract

SARS-CoV-2 can infect domestic animals such as cats and dogs. The zoonotic origin of the disease requires surveillance on animals. Seroprevalence studies are useful tools for detecting previous exposure because the short period of virus shedding in animals makes detection of the virus difficult. We report on an extensive serosurvey on pets in Spain that covered 23 months. We included animals with exposure to SARS-CoV-2–infected persons, random animals, and stray animals in the study. We also evaluated epidemiologic variables such as human accumulated incidence and spatial location. We detected neutralizing antibodies in 3.59% of animals and showed a correlation between COVID-19 incidence in humans and positivity to antibody detection in pets. This study shows that more pets were infected with SARS-CoV-2 than in previous reports based on molecular research, and the findings highlight the need to establish preventive measures to avoid reverse zoonosis events.

Since December 2019, the entire world has experienced the pandemic produced by the novel betacoronavirus SARS-CoV-2. This virus is the causative agent of COVID-19, a severe acute respiratory disease that has resulted in >700 million cases and >6.8 million confirmed deaths (*1*). Although the origin of the virus has not been clarified yet, it is thought to have originated from an animal reservoir and subsequently been transmitted to humans by a direct spillover event (*2*–*4*). Moreover, as the pandemic has progressed, numerous cases of natural SARS-CoV-2 infection have been reported in different animal species, such as minks, cats, dogs, ferrets, nonhuman primates, tigers, and otters (*5*). Most of those cases were associated with exposure to infected humans, a phenomenon defined as reverse zoonosis. This susceptibility is probably due to high homology between the human angiotensin‐converting enzyme 2 (ACE2) receptor and those in several animal species; this receptor plays a key role in the virus entry into the cell (*6*,*7*).

Considering the zoonotic origin of the virus and the ongoing pandemic, both active and passive surveillance should be conducted on animals. Surveillance is particularly important for common pets, such as cats and dogs, because human-to-pet transmission is more likely to occur through close contact between owners and pets. That fact is evidenced by the great number of studies reporting SARS-CoV-2 infection and the presence of antibodies in cats and dogs all over the world (*8*–*14*). Quantitative reverse transcription PCR (qRT-PCR) is used to confirm SARS-CoV-2 infection in animals because of its high sensitivity and specificity (*15*). However, the period of viral shedding in animals is fairly short, according to experimental (*16*) and field (*9*) data; therefore, the detection of viral RNA from pet samples is fortuitous. qRT-PCR–based results confirm the infection by direct detection of the agent; serologic diagnosis may be useful to identify previous exposure in cats and dogs because these species develop a strong antibody-based response to viral infection with SARS-CoV-2 (*17*–*19*). Seroprevalence studies may extend our knowledge about the real prevalence of COVID-19 in pets; it is crucial to use a serologic test with high specificity to avoid cross-reactivity. Thus, the virus neutralization test (VNT) is a recommended technique because it can detect specific neutralizing antibodies against the virus. This assay, in combination with a simpler test for initial serum screening, could obtain specific and reliable results.

In this study, we performed an extensive serosurvey on cats and dogs in Spain, a country that has been severely affected by the COVID-19 pandemic, with >11 million cases and 100,000 thousand deaths (*1*) so far. We performed initial antibody detection using a previously validated screening ELISA (*20*) and posterior confirmation using VNT. The results provide insights into the occurrence of COVID-19 and its spatial distribution in pets throughout the waves of the pandemic. The ethics committee for animal experiments at Complutense University of Madrid approved all the protocols (project license 14/2020).

## Materials and Methods

###  Animal Sample Collection

Practitioners from hospitals, clinics, or Animal Protection Centers (APCs) in Spain collected serum samples from cats (n = 861) and dogs (n = 1,039) in accordance with the guidelines of good experimental practices, following European, national, and regional regulations. Samples were subsequently sent to the Health Surveillance Centre (VISAVET) at the Complutense University of Madrid (Madrid, Spain) by a transport company under the regulations stated in the UN3373 Biological Substance, Category B (*21*), and ARRIVE 2.0 guidelines (*22*). Owners and keepers were duly informed of the purpose of the study and the data protection policy and provided written consent for each pet. Serum samples were collected in tubes without any anticoagulant and kept refrigerated until shipment. At the laboratory, samples were stored at −80°C until analysis. When possible, further samples for qRT-PCR analysis were taken following the methods previously described (*20*).

To avoid a potential sampling bias, the survey included animals with known exposure to persons infected with SARS-CoV-2 as confirmed by qRT-PCR or antigen test, as well as nonexposed animals. We included domestic animals, defined as pets living in houses and animals from APCs (513 cats, 967 dogs), and stray animals, defined as free-ranging dogs or cats captured for sterilization and sampling (304 cats, 54 dogs). The presence or absence of clinical signs compatible with the disease (i.e., respiratory and digestive symptoms, anorexia, and apathy) was recorded for every animal. The study period was January 2020–November 2021. Sampling included animals from 11 autonomous communities in Spain: Andalucía, Aragón, Castilla la Mancha, Castilla y León, Cataluña, Ceuta, Madrid, País Vasco, Valencia, Navarra, and Murcia.

### ELISA Based on RBD

We performed an indirect ELISA test based on the receptor-binding domain (RBD) of the virus as a screening test (Raybiotech, https://www.raybiotech.com). We adapted the ELISA to each species by using a specific anti-species conjugate. In brief, we covered coated plates with 100 μL of 1:40 diluted serum in phosphate-buffered saline (PBS) containing 0.05% Tween 20 (PBS-T) and incubated at 37°C for 30 minutes. We then washed the plates 4 times, added 100 μL of the specific anti-species HRP-conjugated IgG (Jackson Immuno Research Laboratories, https://www.jacksonimmuno.com) diluted 1:18,000 in PBS-T, and incubated the solution at 37°C for 15 minutes. After 4 more washes, we added 100 µL of SureBlue Reserve TMB microwell peroxidase substrate (TMB) (KPL, https://kpl.com) and incubated the plates in the dark for 10 minutes. We stopped the reaction by adding 100 μL of H_2_SO_4_ (3M, https://www.3m.com) to each well. We determined absorbance at 450 nm using an Anthos 2001 plate reader (Labtec, https://anthos-labtec.nl). We determined the endpoint cutoff by the analysis of a receiver operating characteristic (ROC) curve based on positive divided by negative (P/N) values. Validation of this ELISA test was extensively described (*20*).

### Virus and Cells

SARS-CoV-2 MAD6 isolated from a 69-year-old male patient in Madrid, Spain, belonging to the B.1 (Pango v.3.1.162021-11-04) lineage, was provided by Dr. Luis Enjuanes from the National Biotechnology Centre (CNB) at the Higher Council for Scientific Research (CSIC). We prepared Vero E6 cells provided by the Carlos III Healthcare Institute (Madrid, Spain) or ATCC to reproduce the SARS-CoV-2 stocks. We incubated cells at 37°C under 5% CO_2_ in GIBCO Roswell Park Memorial Institute (RPMI) 1640 medium with L-glutamine (Lonza Group Ltd, https://www.lonza.com) and supplemented with 100 IU/mL penicillin, 100 μg/mL streptomycin, and 10% fetal bovine serum (FBS) (Merck KGaA, https://www.emdgroup.com). We determined SARS-CoV-2 titers via a 50% tissue culture infectious dose (TCID_50_) assay.

### VNT for Detection of Specific Neutralizing Antibodies against SARS-CoV-2

We used VNT to confirm the presence of neutralizing antibodies against SARS-CoV-2 in all the samples that showed a doubtful or positive result to the screening ELISA. In brief, we performed the VNT in duplicate in 96-well plates by incubating 25 μL of 2-fold serially diluted serum with 25 μL of 100 TCID_50_/mL of SARS-CoV-2. We incubated the virus/serum mixture at 37°C with 5% CO_2_. After 1 hour, we added 200 μL of Vero E6 cell suspension to the mixtures and incubated the plates at 37°C with 5% CO_2_. We determined the neutralization titers at 5 days postinfection. We recorded the titer of a sample as the reciprocal of the highest serum dilution that provided at least 100% neutralization of the reference virus, as determined by the visualization of cytopathic effect (CPE). We additionally determined cell viability after VNT by using a violet crystal assay to confirm the results observed by microscopy. At the end of VNT (5 days postinfection), we dried the cells, added 200 µL of 0.5% crystal violet solution (Sigma-Aldrich, https://www.sigmaaldrich.com), and incubated the solution at room temperature for 20 minutes. Finally, we removed the crystal violet for the visualization of CPE or cellular tapestry. We determined cell viability by comparing each well with both the virus and the cell control wells.

### Data Analysis

We organized surveillance data on sampled dogs and cats by origin source, date, and results of diagnostic tests against SARS-CoV-2 in all the samples. We structured spatial data at the province level to reduce sampling bias between rural and urban scenarios. We conducted statistical analysis using SPSS Statistics 20 (IBM, http://www.spss.com) and R version 3.5.0 (The R Project for Statistical Computing, https://www.r-project.org). We used dplyr R package (*23*) for database exploration. We performed a descriptive analysis of seroprevalence from ELISA and VNT tests to calculate average ranges per species, sampling groups, and period at 95% CI. We studied variations in these parameters between groups and among different province-periods (3 months per period) with known human incidence of SARS-CoV-2 by a generalized linear mixed model (GzLMM) using a binomial distribution and probit link function. Thus, the response variable of the model was the presence (as 1) or absence (as 0) of a positive case to ELISA and confirmed by VNT test, with the reference value in the binomial distribution. We included sampling location as a random effect factor in the model; we included incidence of SARS-CoV-2 in humans, proportion of stray sampled animals, and contact with >1 person infected with SARS-CoV-2 at the province-period level as independent factors. We applied a protocol for data adjustment and checked the assumptions on the residuals of the model (*24*). We considered outcomes of p <0.05 statistically significant.

## Results

### Detection of Neutralizing Antibodies against SARS-CoV-2

**Figure 1:**
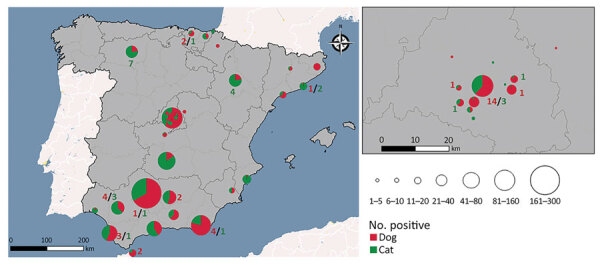
Spatial distribution of sampled animals and those testing positive for SARS-CoV-2 by neutralizing antibodies in study of SARS-CoV-2 seroprevalence studies in pets, Spain. Map at right shows detail of boxed area at left. Red numbers indicate number of positive dogs; green numbers indicate number of positive cats.

A total of 68 samples tested positive to ELISA, and 3 were doubtful samples. We used VNT to determine the presence of specific and neutralizing antibodies against SARS-CoV-2 in those ELISA positive and doubtful (n = 71) samples from cats and dogs. Positive results were confirmed in 66/71 ELISA-positive samples (ELISA specificity = 92.95) (Table 1). A total of 66 animals (3.59% of the total) showed neutralizing antibodies, 28 cats (seroprevalence of 3.43%), and 38 dogs (seroprevalence of 3.73%) (Figure 1). Overall, 60 positive cases were domestic animals whereas 6 stray animals resulted positive. All the stray animals that showed neutralizing antibodies were cats. Out of the 66 positive animals, 44 had contact with >1 person infected with SARS-CoV-2; 16 of those also had symptoms compatible with the infection, including sneezing, cough, and diarrhea. Out of the 60 positive domestic animals, 44 were pets living in houses, and 16 were housed in APCs. Six animals that showed neutralizing antibodies were also positive by qRT-PCR. VNT titers varied among samples; the lowest recorded was 1:32 and the highest 1:256. We observed no statistical differences in the VNT titers for cats and dogs.

**Table 1:** Distribution of cats and dogs testing positive for SARS-CoV-2 by location and type, Spain*

Autonomous community	No. cats infected/total			No. dogs infected/total		Total no. animals
	Domestic	Stray		Domestic	Stray	
Andalucía	6/305	0/89		15/616	0/2	21/1,012
Aragon	0/19	3/36		0/2	0/13	3/70
Cataluña	2/27	0/6		2/35	0/4	4/72
Castilla La Mancha	0/2	2/117		0/16	0/14	2/149
Castilla y León	7/37	0/13		0/14	0/1	7/65
Ceuta	0/5	0/0		2/19	0/0	2/24
Madrid	6/93	0/12		16/236	0/18	22/359
Murcia	0/7	0/0		0/8	0/0	0/15
Navarra	0/0	0/0		0/3	0/0	0/3
País Vasco	1/17	0/0		3/18	0/0	4/35
Valencia	0/1	0/31		0/0	0/0	0/32
Total no. animals	27/817			38/1,019		65/1,836

*Animal origin is domestic (animal living in a house or housed in Animal Protection Centers) or stray (free-ranging animals captured for sterilization and sampling). The numbers shown correspond to the number of animals positive by virus neutralization test-with respect to the total number of animals sampled.

### Factors Explaining SARS-CoV-2 Seroprevalence in Pets

**Figure 2:**
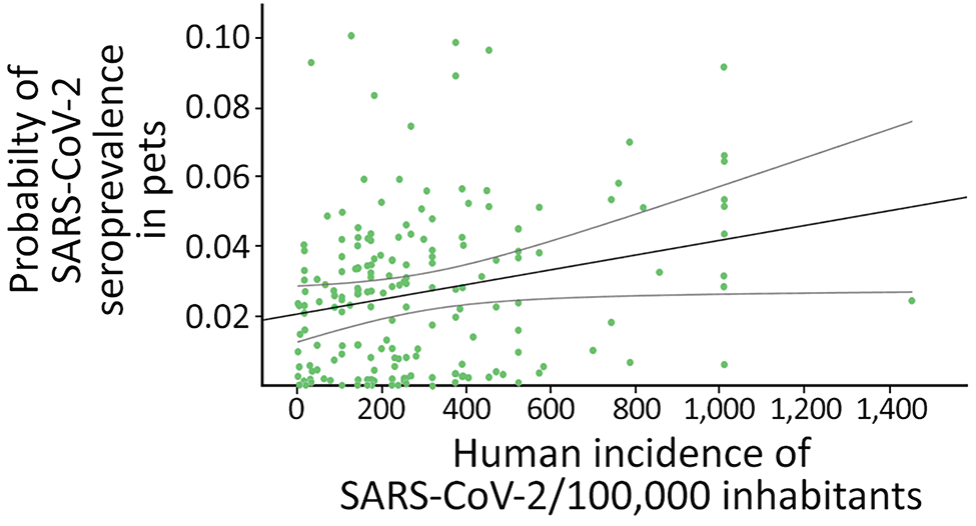
Predicted probability of SARS-CoV-2 seroprevalence in pets as related to registered human incidence (cases per 100,000 inhabitants) at the province-period (3 months each period) level in study of SARS-CoV-2 seroprevalence studies in pets, Spain. The black line marks the trend and slope of the correlation. Lighter gray lines show 95% CIs.

When we considered the GzLMM on the seroprevalence variations obtained by VNT, we observed statistical differences once controlled by other factors, such as sampling locations and periods among groups (Table 2). The overall risk for SARS-CoV-2 seroprevalence in pets increased proportionally to the human incidence of this pathogen (β = 4.85; p<0.001) (Figure 2). In fact, we observed a higher risk for seroprevalence in animals with previous contact with >1 positive person (β = 8.23; p<0.001). The risk for SARS-CoV-2 seroprevalence in stray animals was significantly lower than in domestic animals (β = −4.63; p<0.001).

**Table 2:** Variations of SARS-CoV-2 seroprevalence in pets by human contact, Spain*

Model terms	Estimate	SE	Z value	p value
Intercept	−1.274	0.240	−5.307	<0.001
Human incidence	0.001	0.001	4.852	<0.001
Contact	0.116	0.014	8.234	<0.001
Stray	−0.058	0.012	−4.628	<0.001

*Generalized linear mixed model output to explain variations of SARS-CoV-2 seroprevalence in pets in relation to the officially registered human incidence of SARS-CoV-2, the proportion of animals with previous contact with >1 positive person (Contact), and the proportion of stray animals. Sampling location was considered a random effect factor.

## Discussion

Since the beginning of the COVID-19 pandemic, many studies have shown that pet cats and dogs are susceptible to SARS-CoV-2 infection, both experimentally (*16*) and naturally (*18*,*25*). Active infection in pets triggers the development of an effective immune response based on neutralizing antibodies, as previously demonstrated (*17*,*19*). Positivity by PCR tests lasts as long as the active infection does, 5–17 days (*19*,*26*). This short period in which positive PCR results are obtained hinders the detection of minor infections that typically occur in pets. In contrast, antibodies persist in serum for longer periods, <28 weeks (*27*), which makes those tests a helpful tool for evaluating previous exposure to the disease. Here, we evaluated a large number of samples from cats and dogs in Spain during a 23-month period, demonstrating a higher rate of antibody positivity than in previous seroprevalence studies. As we expected, the risk for SARS-CoV-2 seroprevalence in stray animals was lower than that for domestic animals. We have observed that seropositivity in animals increased proportionally to the human incidence of SARS-CoV-2. Therefore, the epidemiology of the disease in the human population has an effect on animal seroprevalence.

Taking into account the current state of the COVID-19 pandemic, we cannot rule out changes in the epidemiology of the disease. As new variants emerge, SARS-CoV-2 can adapt to other hosts such as cats and dogs. Clarifying the distribution of the disease in cats and dogs can reveal infection trends in these species.

This study has several strengths in addition to the large number of samples analyzed. We have monitored both animals in contact with SARS-CoV-2–infected persons as well as animals with no previous exposure to the disease; we also analyzed a high number of samples from stray animals, which can give us information about infections caused by environmental contamination and virus circulation in the field. Moreover, the period of our study was long, which enabled us to evaluate infection trends in pets during the different waves in the human population. Because the study ended in November 2021, our results reflect the seroprevalence triggered by 3 consecutive variants of SARS-CoV-2: B.1, Alpha, and Delta. According to the sequencing reports from the government of Spain, B.1 variant was the most prevalent strain during March 2020–March 2021. More recently, the Alpha variant became dominant in the country until September 2021, the point in which the Delta variant replaced Alpha. The Omicron variant was introduced in Spain in December 2021, so no information about seroprevalence during the Omicron wave was available for this study. We note that the viral strain used for VNT in this study was the B.1 strain. The specificity of antibodies against this variant may have influenced the titers of neutralizing antibodies obtained from serum samples during subsequent waves, and, therefore, underestimated the level of antibodies in some cases.

The percentage of seropositivity in this work was slightly higher (3.56%) in comparison with other studies with similar sample size, as previously described in the United States (0.17%) (*28*), Italy (4.04%) (*10*), Germany (0.43%) (*29*), and the Netherlands (0.3%) (*30*). This finding could be related to the high COVID-19 incidence in humans in Spain during the study period. In January 2021, accumulated incidence reached ≈900 positive/100,000 inhabitants in Spain, followed by a few months in which the accumulated incidence exceeded 100 positive/100,000 inhabitants until July, when another peak was reached (700 positive/100,000 inhabitants). Subsequently, accumulated incidence had a large drop and remained <100 positive/100,000 inhabitants during September–November 2021. As demonstrated by the GzLMM, the incidence of SARS-CoV-2 infection in the human population was related to higher positivity to VNT in pets. This finding highlights the importance of taking preventive measures and minimizing contact with domestic animals when humans become infected. Because the epidemiologic scenario of the disease may change at any time due to the high rate of genomic mutation of the virus and the apparition of new variants, it is crucial to limit the contagion of susceptible species.

Previous studies on pets in Spain have demonstrated a low prevalence of positive animals by PCR (*11*,*20*). However, as we have demonstrated, more animals have been exposed to the virus. In all those cases, we can confirm that the exposure resulted in an active infection because the animals were able to develop an effective immune response based on neutralizing antibodies. We suspect that SARS-CoV-2 infection in pets is anecdotic because in none of the positive cases we described did the owners detect severe symptoms in the animals. Although some animals had antibodies and were experiencing clinical signs at the time of sampling (30.3%), such as sneezes, dyspnea, nasal discharge, coughs, vomiting, or depression, the relationship between those signs and the SARS-CoV-2 infection is not clear enough. Antibodies remain undetectable in serum until 8–10 days postinfection (*19*), leading to a delay between a positive result to antibody detection and the infection. In addition, a high percentage of the animals were sampled during their attendance at the veterinary clinic, and the symptoms reported as the reason for the visit might be unrelated to SARS-CoV-2 infection; comorbidities may cause a biased result.

We confirmed that stray animals had neutralizing antibodies, as do domestic animals in contact with SARS-CoV-2–infected persons. Those results are in line with those from other studies that confirmed the presence of neutralizing antibodies in stray animals (*31*–*33*). However, the seroprevalence in this group of animals was very low; the domestic animals represented 4.05% of the animals with neutralizing antibodies, compared with 1.69% in the case of stray animals. Those results make sense because domestic animals are more likely to be in contact with infected persons and share potentially contaminated spaces than stray animals. In those cases, the exposure to the virus may be related to the times that humans fed the stray colonies and to the presence of infectious excretions in the areas frequented by stray cats and dogs. Another potential route of transmission is animal-to-animal transmission, which has been demonstrated in the case of stray cats (*9*). These results suggest that virus circulation in stray populations is low, although special care should be taken in practices that may pose a risk, such as the feeding of stray animals.

In conclusion, this study demonstrated higher rates of human-to-pet SARS-CoV-2 transmission than those found by direct molecular detection. As expected, the seroprevalence of the disease was higher in animals with previous exposure to infected persons, whereas the lower risk of infection in stray animals is likely caused by a low rate of exposure. In addition, the epidemiology of the disease in the human population seems to influence the seroprevalence of the infection in cats and dogs, which highlights the importance of performing active surveillance in susceptible species.
